# Porous Waterborne Polyurethane Films Templated from Pickering Foams for Fabrication of Synthetic Leather

**DOI:** 10.1021/acs.langmuir.3c03514

**Published:** 2024-02-22

**Authors:** Zhenghao Shi, Yifeng Sheng, Jianhui Wu, Jiwei Cui, Wei Lin, To Ngai

**Affiliations:** † Department of Chemistry, The Chinese University of Hong Kong, Shatin, Hong Kong 999077, China; ‡ Department of Biomass and Leather Engineering, Key Laboratory of Leather Chemistry and Engineering of Ministry of Education, Sichuan University, Chengdu 610065, China; § Key Laboratory of Colloid and Interface Chemistry of the Ministry of Education, School of Chemistry and Chemical Engineering, Shandong University, Jinan, Shandong 250100, China

## Abstract

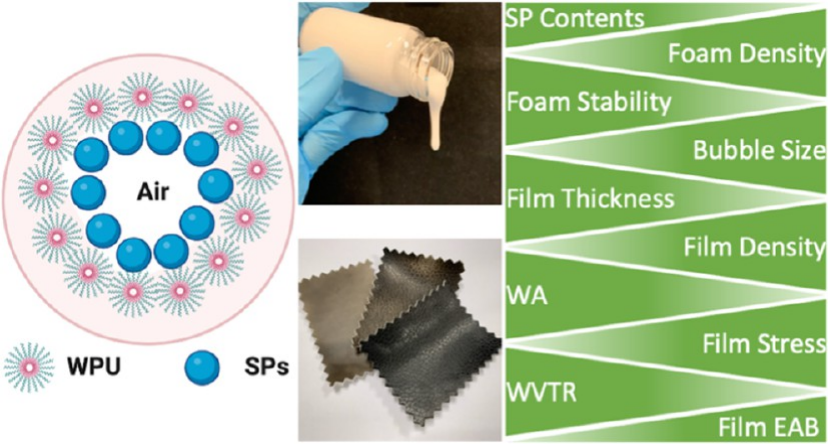

Waterborne polyurethane (WPU) latex nanoparticles with proven interfacial activity were utilized to stabilize air–water interfaces of Pickering foams through interfacial interaction with hydrophobic fumed silica particles (SPs). The rheological properties of the Pickering foam were tailored through adjustment of their SP content, which influenced their formability and stability. A Pickering foam stabilized with WPU and SPs was used as a template to prepare a WPU–SP composite porous film. The as-prepared film had intact open-cell porous structures, which increased its water absorption and water-vapor permeability. The porous film was used as a middle layer in the preparation of synthetic leather via a four-step “drying method”. Compared with commercial synthetic leather, the lab-made synthetic leather with a middle layer made of the WPU–SP composite porous film exhibited a richer porous structure, acceptable wetting on a fabric substrate, a thicker porous layer, and higher water-vapor permeability. This work provides a novel and facile approach for preparing WPU–SP Pickering foams. Furthermore, the foams have the potential to function as a sustainable material for creating a porous-structured synthetic leather made from WPU, which may be utilized as an alternative to solvent-based synthetic leather.

## 1 Introduction

Leather is a unique commodity with a market value that is estimated to reach 360 billion USD by 2025. It links rural farmers and factories to the fashion world, as it is warm but breathable and strong but flexible.^1,2^ However, the leather industry produces a significant amount of tannery wastes, including wastewater, hazardous chemicals, and gaseous pollutants.^2−4^ The reliance on polluting, hazardous, and toxic chemicals in current manufacturing systems is unsustainable. Products, feedstocks, and manufacturing processes are being incorporated with sustainable techniques, such as the use of green chemistry and green engineering.^5,6^ The manufacturing of synthetic leather, also known as artificial leather, which has a leather-like appearance and serves as a substitute for natural leather, is shifting from the use of fossil-fuel-based chemicals such as polyurethane (PU)^7,8^ and poly(vinyl chloride) (PVC)^9,10^ to the implementation of more environmentally friendly techniques. Civil and industrial wastes such as leather solid waste,^11^ pineapple leaf,^12,13^ jute fiber,^13^ coffee grounds,^14^ and bagasse^15^ are being adopted for synthetic leather production; moreover, bioderived materials such as mycelium,^2,16^ cellulose nanocrystals,^17^ and bacterial cellulose^18,19^ are being used to promote environmental sustainability.

PU has emerged as the predominant material in the manufacturing of synthetic leather. In 2021, PU-based leathers constituted more than 55% of the industry’s worldwide sales.^20^ PU-based leathers provide a comparable tactile sensation to natural leather owing to the resemblance between the urethane group in PU and the peptide chain in collagen. In addition, as compared to genuine leather, PU features enhanced waterproof properties and is softer and lighter but has poorer breathability (i.e., less water-vapor permeability).^21,22^ The leather business, including the PU-based synthetic leather industry, has been compelled to create eco-friendly production techniques and products due to advancements in living standards and increasing consumer appetite for luxury goods, especially in the automobile and footwear sectors.

Waterborne polyurethane (WPU) has emerged as an environmentally friendly industrial raw material by means of hydrophilic group introduction onto hydrophobic PU chains. This process facilitates the uniform dispersion of PU in aqueous media by employing tertiary amines (cationic WPU), carboxylic acids (anionic WPU), and sulfonic acids (anionic WPU) as hydrophilic groups.^23^ The hydrophobic part of WPU molecules aggregates in aqueous media to form the core of WPU latex particles, while the hydrophilic ionic groups extend out on the surface of each of these particles.^24^ Researchers and enterprises have shown considerable interest in using water instead of organic solvents in the manufacturing process of PU products. Moreover, WPU has gained considerable attention owing to its nontoxicity, odorlessness, convenient storage properties, safety, and environmental friendliness.^21^ Nevertheless, WPU-based synthetic leather manufactured via phase inversion features a lower water-vapor permeability than solvent-based PU coatings.^25,26^ Porous structures prepared using a physical blowing agent^27^ or via particle templating (i.e., solvent casting and particle leaching)^28^ and emulsion templating^29^ have demonstrated promising water-vapor transmission abilities, but such methods are no longer favored in the leather industry owing to restrictions on the use of volatile organic compounds (VOCs).

The use of wet foam as a template for preparing porous materials was suggested because the approach is organic solvent-free in nature and processing-friendly.^24^ Nevertheless, because the desorption energies of surfactants are comparable to their thermal energies, their air–water interfaces are prone to undergoing constant adsorption and desorption of surfactants. Consequently, this leads to interfacial instability and the formation of uncontrolled pore shapes and sizes.^30^ The process of chemical bond formation, such as polymerization or cross-linking the continuous phase of a wet foam, can solidify a wet foam to be a template to produce porous materials.^31^ However, it is difficult to apply this technique to the continuous production of synthetic leather. Particle-stabilized foam (i.e., Pickering foam) can remain stable for many months. It has found applications in several sectors such as mineral^32^ and polymer industries.^33^ Additionally, it is highly regarded as an excellent template for creating porous materials.^34,35^ Contrary to wet foams sustained by surfactants, Pickering foams can limit Ostwald ripening, coalescence, and disproportionation during drying. This is because the particles irreversibly adhere to the air–water interface.^36,37^ Hydrophilic particles, with water contact angles of 60–70°, are considered ideal stabilizers for Pickering foams, whereas hydrophobic particles are considered defoamers.^30,34,38^ A recent study has demonstrated that particles with higher hydrophobicity improve the aqueous foam stability by cooperating along with other hydrophilic components at the air–water interface during the formation of binary component stabilized Pickering foam.^39^ Despite Pickering foam exhibiting superior stability in comparison to other types of foam, the drainage inside the foam’s continuous phases causes the liquid thin film among bubbles to become weaker, finally resulting in the rupture of the foam bubbles.^26^ Therefore, it is crucial to solidify the continuous phase of a Pickering foam in order to establish it as a template for achieving a highly porous structure.^37^ A gel-like continuous phase has been created by establishing a physically interconnected network between hydrophobic silica particles (SPs) and cellulose nanofibers, which effectively prevents the wet Pickering foam from collapsing upon drying.^40^

**Figure 1:**
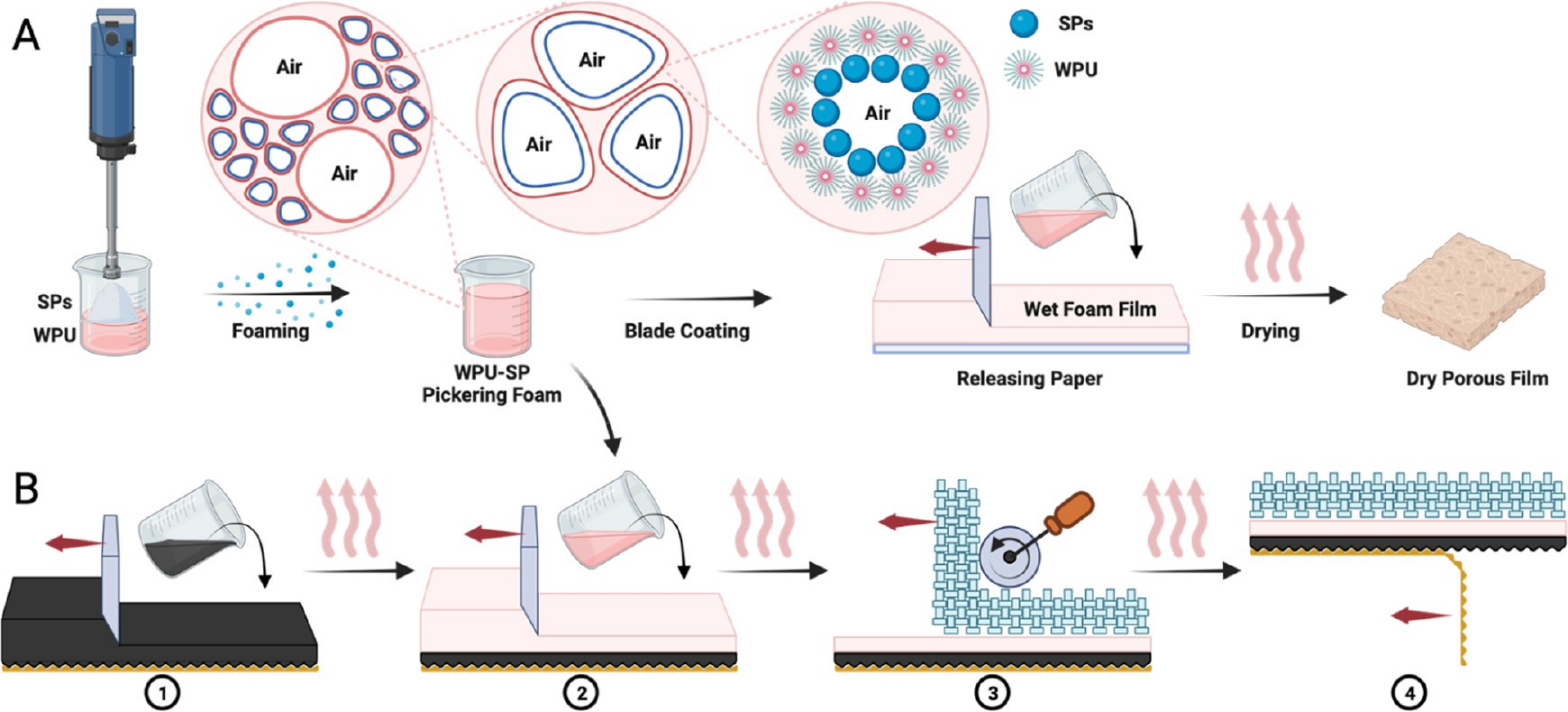
(A) Procedure of preparing a WPU–SP composite porous film involves mechanical foaming, followed by blade-coating and drying. (B) Diagram depicts a four-step “drying method” employed in the production of synthetic leather, which comprises (1) surface layer preparation, (2) porous-layer preparation, (3) fabric binding, and (4) peeling the film off the releasing paper.

To develop a clean production method for fabricating WPU-based synthetic leather with improved water-vapor permeability, a binary particle-stabilized Pickering foam was prepared from WPU and hydrophobic silica nanoparticles. The air–water interface of the wet foam was stabilized by the WPU and SPs. Specifically, a series of WPU–SP Pickering foams (with SP loading: 0–8 wt %) were prepared via mechanical frothing following the scheme shown in Figure 1A. The wet film, regulated to a thickness of 1000 μm using a blade coater, was applied and dried on releasing paper. It was then used as a template to fabricate porous WPU–SP composite films. The porous WPU–SP composite films were ultimately utilized as the middle layer in the production of synthetic leather (Figure 1B).^22^ The physicochemical properties of the WPU–SP Pickering foams, including their stability, interfacial structures, and rheological properties, were investigated. The water-vapor transmission rates (WVTRs), one of the key performance indicators of synthetic leather, of the porous films and lab-made synthetic leather were investigated. The utilization of WPU–SP stabilized Pickering foams in the creation of porous films offers a unique approach to boosting the water-vapor permeability of WPU synthetic leathers. This advancement has the potential to enable clean and sustainable manufacturing in the synthetic leather industry.

## 2 Experiments

### 2.1 Materials

Commercial anionic WPU samples: WPU1 (PU700A, particle size = 35 nm) was purchased from Xinmiao Chemical Co., Ltd., and WPU2 (XWB8016, particle size = 350 nm) and WPU3 (XWB4260) were purchased from Xuchan Chemical Co., Ltd. WPU1 was used to prepare most of the WPU–SP Pickering foams, while WPU2 was used for interfacial structure analysis. WPU3 mixed with a black pigment (10 wt %) was used to prepare the surface layer for synthetic leather. Commercial hydrophobic fumed silica nanoparticles (HDK H18, with a specific surface area of 170–230 m^2^/g) were purchased from Wacker Chemie. Perylene (99%) was provided by Acros Organics. Nile red was purchased from Aladdin Biochemical Technology. Ethanol (AR grade) was purchased from Fisher Scientific. Calcium chloride dihydrate (94%, 1–3 mm) was purchased from Maclin Biochemical Co. Ltd. Toluene (GR grade) was obtained from Duksan Reagents Co. Ltd. Deionized water (18.2 MΩ·cm) prepared from a Smart 2 Pure Millipore water system (Thermo Scientific, Sweden) was used in all of the experiments. All of the chemicals were used without further purification unless stated otherwise.

### 2.2 Preparation of Fluorescent WPU and SPs

To investigate the air–water interface structures of the bubbles costabilized by WPU latex particles and hydrophobic SPs, WPU and SPs were labeled with different fluorescent dyes. The SPs were labeled with perylene, a hydrophobic fluorescent dye, via physical adsorption. Specifically, SPs (5 g) were dispersed in toluene (50 mL) under sonication for 30 min and then 0.05 g of perylene was added to the mixture.^37^ The fluorescent SPs were collected via centrifugation, washed by toluene three times, and finally dried in a vacuum oven to constant weight. The WPU latex particles, consisting of a hydrophobic core and a hydrophilic shell, were marked with Nile red, a hydrophobic fluorescent dye. Specifically, Nile red (0.5 mg) was allowed to float on the surface of WPU (50 mL). Then, Nile red-labeled WPU latex particles were collected via mechanical frothing to ensure that the Nile red was well dispersed in the aqueous media and thus adsorbed to the hydrophobic core of the latex particles. The Nile red-labeled WPU was subjected to degassing at room temperature in a vacuum oven to get ready for the Pickering foam air–water interface properties study.

### 2.3 Preparation of WPU–SP Pickering Foams

A series of WPU–SP Pickering foams were fabricated via a one-step foaming process using normal or fluorescent dye-labeled WPU and SPs (Figure 1A). The static contact angles of a 5 μL droplet on the SPs were measured using a KRÜSS DSA30b (KRÜSS GmbH, Hamburg, Germany; Figure S1). The foaming process was conducted at ambient conditions, i.e., 25 °C and 40–60% relative humidity (RH). SPs were added to 20 g of WPU at concentrations of 2, 4, 6, and 8 wt % and allowed to float on the WPU surface to give samples of WPU–SP2, WPU–SP4, WPU–SP6, and WPU–SP8, respectively. The sample without SP was denoted “WPU–SP0”. Each liquid–particle mixture was frothed with Ultra-Turrax (Ika digital T25, IKA-Werke GmbH & Co. KG, Staufen, Germany) equipped with an S25N-18G stainless steel dispersing tool at speeds of 3000–6000 rpm for 3 min. The homogenizer tip was first positioned in the air–liquid–particle mixture to introduce particles and air into the liquid. The tip was immersed into the liquid and alternately moved from the surface to the bottom of the mixture, thereby ensuring that the particles were entirely drawn into the liquid phase. The resulting Pickering foams consisted of a continuous aqueous phase containing hydrophilic WPU latex particles and a dispersed air phase containing hydrophobic SPs. WPU and SPs exhibited interaction and adhesion with one another at the air–water interface.

### 2.4 Characterization of WPU–SP Pickering Foams

As-prepared Pickering foams were transferred into 30 mL sample tubes and tightly sealed for the purpose of evaluating their foamability and tracking their foam stability within 30 days after homogenization. Their stability was determined in terms of the drainage occurrence time and volume changes in the liquid–foam layer. Foamability was described in terms of the foam index (FI), which is the percentage ratio of the foam volume after the foaming process to that before the homogenization process (eq 1).^40^ A greater FI value indicates a higher foamability.
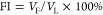
1where *V*_F_ represents the volume of the Pickering foam and *V*_L_ represents the volume of the liquid WPU before the foaming process.

A fluorescent Pickering foam (WPU containing 2 wt % of fluorescent SPs) was prepared to evaluate the binary particle-stabilized air–water interfacial structure of the wet foam. Specifically, the fluorescent wet foam was investigated via confocal scanning laser microscopy (CSLM) (40×, ECLIPSE C1si, Nikon Co. Ltd., Tokyo, Japan). Perylene and Nile red were excited with lasers of wavelengths 408 and 543 nm, respectively. Wet Pickering foam bubble size changes were tracked via CSLM (4×/20×) within 24 h after the foaming process. Normal Pickering foam samples used for size change tracking were placed in the center of a rubber ring (20 mm in diameter and 2 mm thick) clamped between two pieces of glass and tightly sealed to ensure the wet status of Pickering foam samples during the observation. The bubble size at each time point was determined by computing the mean diameter of a minimum of 200 bubbles. A rheometer (Malvern Kinexus Lap+, Malvern Instruments Inc., U.K.) equipped with 20 mm parallel plates was used to measure the bulk rheological properties of the Pickering foams. In the oscillatory shear measurement, the time sweep, frequency sweep, amplitude sweep, and gap were set at 120 s, 0.1%, 0.1–100 rad s^–1^, and 0.9 mm, respectively, for each sample. All of the Pickering foam samples used for optical and rheological measurements were acquired from the central region of wet foam body, and the experiments were performed at a temperature of 25 °C.

### 2.5 Preparation and Characterization of WPU–SP Composite Porous Films

A series of WPU–SP composite porous films with varying SP loadings were prepared using the Pickering foam which was achieved beforehand as a template (Figure 1A). Pickering foams were applied onto the releasing paper using a blade-coating method to create a thin wet film, with the film thickness carefully regulated at 1000 μm. The as-prepared wet films were dried in the fan oven at 100 °C to constant weight, and porous films were achieved after the releasing papers were peeled off. The porous morphologies of film surfaces and cross sections were assessed via an FEI Quanta 400F field-emission scanning electron microscope (SEM) (Hillsboro, OR). The water affinity of the porous films and their suitability as the midlayer in synthetic leather were determined using water absorption (WA) and WVTR analysis. WA investigations were performed by submerging sample films (round pieces with a diameter of 20 mm) in deionized water for 24 h (eq 2). WVTR analyses were conducted using headspace bottles filled with calcium chloride, which served as a water absorbent. Sample films were positioned over the opening of the bottle and securely fastened with the cap. A 10 mm orifice in the bottle cap was utilized for the purpose of water-vapor transmission. Calcium chloride pellets were predried at 150 °C for 3 h and kept 5 mm away from the sample film. All of the WVTR samples were placed in a humidity chamber (HWS-50B, Shanghai Kuntian Instrument Co., Ltd.) at 90% RH and 40 °C for 24 h, in accordance with the industrial standards (QB/T 1416–2007 and JIS.K6601–1995). The mechanical characteristics, including the tensile strength (TS) and elongation at break (EAB), were assessed. The sample films were trimmed into dumbbell-shaped strips (60 mm × 5 mm, effective length × width) following the GB/T 8949–2008 (China) standard. Subsequently, these strips were subjected to testing with a universal tensile testing instruments (TOHNICHI, Zhuoyue, Dongguan, China) equipped with a 1 kN load cell capacity.
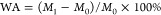
2where *M*_0_ and *M*_1_ represent the mass of the sample film before and after immersion in deionized water for 24 h, respectively
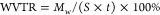
3where *M*_w_ represents the mass of water absorbed by calcium chloride after a typical testing period and *S* and *t* represent the effective area of the sample film through which water vapor can penetrate and the testing duration, respectively.

### 2.6 Preparation and Characterization of Synthetic Leather Based on WPU–SP Composite Porous Films

Synthetic leather features a multilayered sandwich structure, comprising a surface layer, a middle porous layer, and a piece of fabric. The fabric serves as the substrate, and the surface layer provides color, pattern, and some other properties. Tactile sensation and water-vapor permeability are primarily influenced by the middle layer, which was improved by the WPU–SP composite porous films. The lab-made synthetic leather was prepared in four steps (Figure 1B).^22^ The surface layer was formed by blade-coating in spreading a black pigment containing WPU3 onto the releasing paper, resulting in a wet foam with a precisely regulated thickness of 200 nm. The surface layer was then dried in a fan oven until constant weight. Leather patterns could be transferred from the releasing paper, creating typical leather patterns on the synthetic leather surface. The WPU–SP Pickering foams with varying SP contents (0, 2, 4, 6, and 8 wt %) were blade-coated onto the dry surface layer to give a wet film with a thickness around 1000 μm and then dried in a fan oven at 100 °C. Adhesive was not used in binding fabrics to the middle porous layer. Instead, following a 5 min drying period, the fabric was placed onto the partially dried porous layer and further compressed using a roller to achieve thorough penetration of the WPU–SP Pickering foam onto the fabric fibers. The assembled semidried synthetic leather was placed back into the oven and dried at 100 °C until constant weight. Then, the releasing paper was peeled off to obtain the lab-made synthetic leather samples of Lea-WPU–SP0/2/4/6/8, respectively. The porous structures and porous layer thickness of the lab-made synthetic leather were evaluated via FE-SEM. WA and WVTR were determined following the method described in Section 2.5. To illustrate the viability of utilizing the WPU–SP composite porous film as the midlayer in synthetic leather, the corresponding properties of three commercial synthetic leather samples were also examined.

## 3 Results and Discussion

### 3.1 Stability and Foamability of WPU–SP Pickering Foams

**Figure 2:**
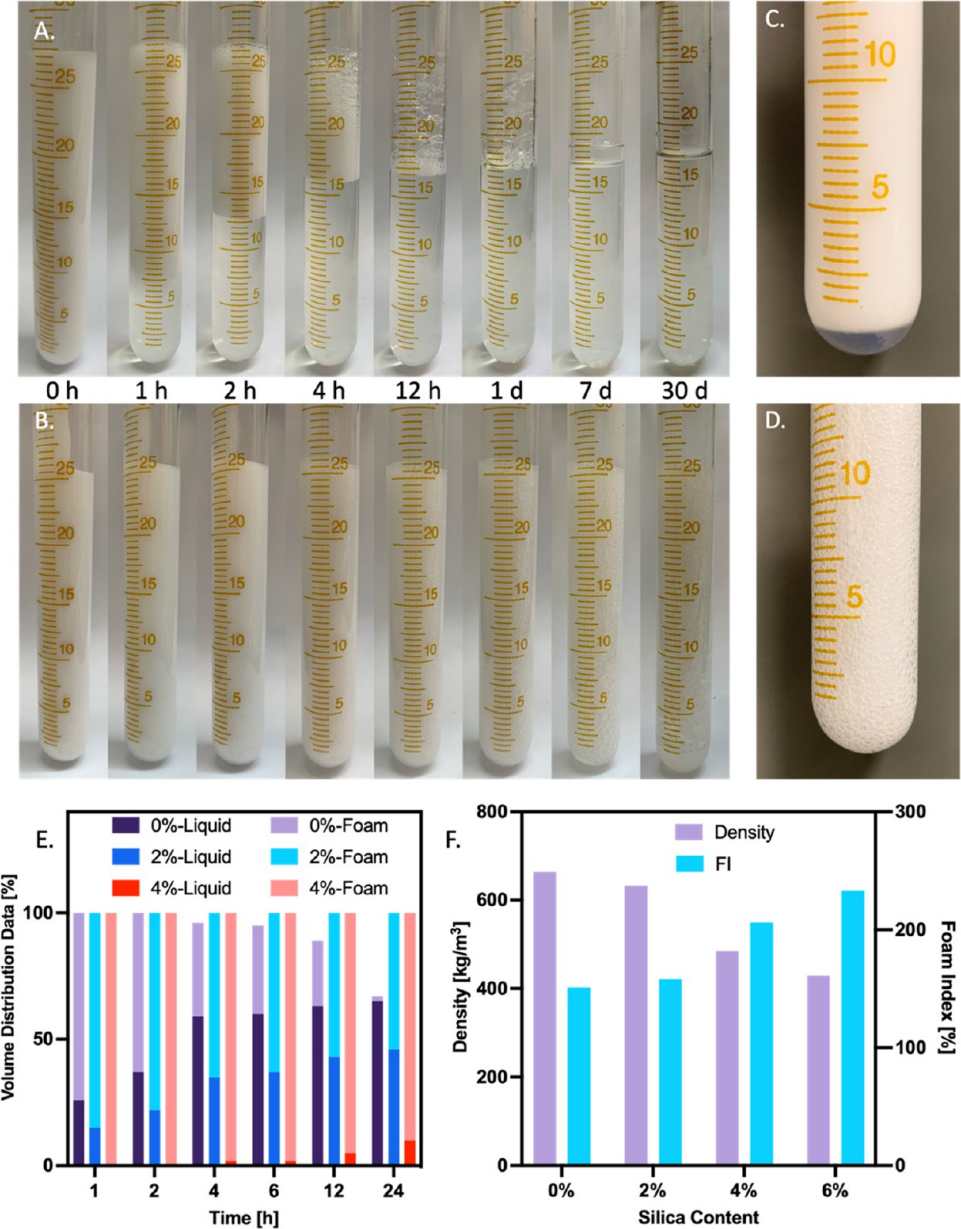
Volume changes in foam and liquid in (A) WPU–SP0 and (B) WPU–SP6. (C) Drainage in the pure WPU–SP0 sample 5 min after foaming. (D) WPU–SP6 exhibited no drainage even 24 h after foaming. (E) Volume of the foam and drainage liquid of WPU–SP0, WPU–SP2, and WPU–SP4 over 24 h. (F) Wet foam densities and FI values of the WPU–SP0, WPU–SP2, WPU–SP4, and WPU–SP6 wet foams.

A previous study showed that the air–water interface could be stabilized with poly(vinyl alcohol) (PVA) molecules and hydrophobic SPs through the formation of a Janus bilayer structure. The achieved PVA-SP Pickering foam could be utilized as a template to produce porous materials.^37^ The objective of this research work was to produce PU porous films, so WPU was employed as a substitute for PVA. Pure WPU-based wet foam is not stable, even for a short period. Thus, WPU–SP0 exhibited drainage just 5 min after foaming (Figure 2C); over 90% of the WPU liquid leaked from the foam layer 2 h after foaming, and most of the foam structure underwent rupture 24 h after foaming (Figure 2A). Incorporating hydrophobic SPs to the WPU Pickering foams enhanced the Pickering foam stability. WPU–SP2 and WPU–SP4 showed a delay in drainage, occurring at 1 and 4 h after foaming, respectively (Figures S2 and S3). Even 24 h after frothing, WPU–SP6 demonstrated no discharge; the only evident change was an increase in bubble size (Figure 2B,D). Over the 7-day observation, all of the WPU–SP Pickering foam samples maintained excellent structural stability with no significant volume loss. WPU–SP6 demonstrated a mere 1% of drainage (Figure 2B and Table S1). The wet foam density and FI values of all of the wet foam samples immediately after foaming are summarized in Figure 2F. When comparing Pickering foams with lower SP contents to those with greater SP contents, it was shown that the foams with higher SP contents had better foamability and therefore lower wet foam densities. WPU–SP2, WPU–SP4, and WPU–SP6 demonstrated considerably higher FI values (>230%) than the pure WPU wet foam (151%). WPU–SP0 exhibited the highest density 663.88 kg/m^3^, followed by WPU–SP2 (633 kg/m^3^) and WPU–SP4 (486 kg/m^3^). WPU–SP6 demonstrated the greatest foamability (233%) and the lowest density (429.29 kg/m3). These consistent and abrupt alterations brought about by the addition of SPs are attributable to increases in Pickering foam stability, owing to interactions between WPU latex particles and SPs.

**Figure 3:**
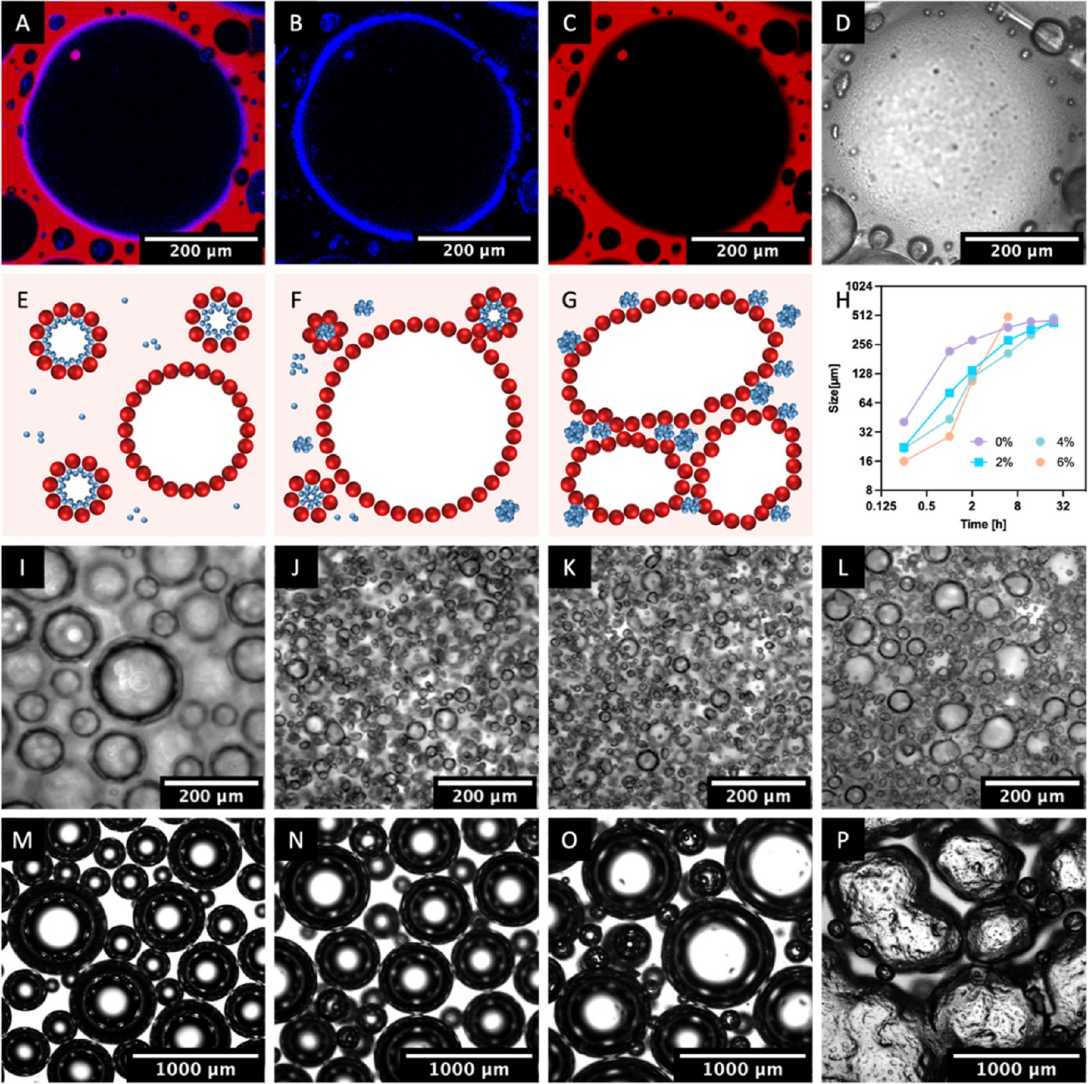
Confocal graphs of WPU–SP Pickering foams made from Nile red-labeled WPU and perylene-labeled SPs under excitation by lasers of wavelengths (A) 408 and 543 nm, (B) 408 nm only, and (C) 543 nm only; and (D) a bright-field image. Schematic of the coarsening process of the Pickering foam bubbles (E–G). The average bubble sizes of WPU–SP0, WPU–SP2, WPU–SP4, and WPU–SP6 at different time points after foaming (H). Bubble aging performance in terms of bubble size 15 min after foaming for (I) WPU–SP0, (J) WPU–SP2, (K) WPU–SP4, and (L) WPU–SP6 and 12 h after foaming for (M) WPU–SP0, (N) WPU–SP2, (O) WPU–SP4, and (P) WPU–SP6.

### 3.2 Aging Process of WPU–SP Pickering Foams

The WPU–SP Pickering foams prepared through the methods described in Section 2.3 were mixtures of water, WPU latex particles, SPs, and air. The confocal graphs depicted in Figure 3A–D show the structure of fluorescent WPU–SP Pickering foams made from Nile red-labeled WPU (red) and perylene-labeled SP (blue). The bright blue ring mapped out of the continuous red area indicates that the SPs remained on the inner wall of the WPU–SP Pickering foam air bubble. While some aggregates of SPs were observed in the continuous phase, it suggests that the majority of the SPs served as Pickering foam stabilizers immediately following foaming (Figure 3B).

The above-mentioned results prove that SP addition can effectively improve the stability of a WPU wet foam. The coarsening and disproportionation processes of the air bubbles of a WPU–SP Pickering foam are illustrated in Figure 3E–G. Two kinds of air bubbles, namely, Pickering foam bubbles stabilized by numerous binary particles (WPU and SPs) and pure WPU-stabilized bubbles, were generated through the introduction of the air phase into the aqueous media after foaming. During the aging process, air in the smaller size Pickering foam bubbles penetrated through the thin liquid film into the larger pure WPU bubbles, owing to Ostwald ripening.^41^ Consequently, the WPU–SP bubbles decreased in size, while the pure WPU-stabilized bubbles continuously absorb the air from the WPU–SP bubbles leading to dimension increase, thereby accelerating the drainage of the liquid phase. Drainage reduced the distance between bubbles, which further accelerated the shrinkage of the WPU–SP bubbles and caused the bubbles to evolve into SP aggregates after air was exhausted. The SP aggregates were wetted by WPU and dispersed in the continuous liquid phase,^42^ which increased the liquid phase viscosity, restricted liquid flow, mitigated the drainage effect, and increased the wet foam structural stability. Once the proportion of SPs dispersed in WPU was great enough to restrict the movement of the WPU–SP Pickering foam, the resulting SP aggregates adhered to the air–water interface of pure WPU bubbles during bubble expansion. Consequently, the air–water interface of SP-aggregate-reinforced bubble exhibited enhanced mechanical properties, facilitating the bubbles’ progressive expansion and transformation into a “rock-like” morphology.

Changes in bubble sizes (15 min, 1, 2, 6, 12, and 24 h after foaming, respectively) were investigated via CSLM to elucidate the coarsening and disproportionation processes of the wet foam bubbles (Figure 3H). WPU–SP0 exhibited the largest bubble size (40.5 nm) 15 min after foaming (Figure 3I). The foam bubbles formed a regular ball structure and repelled each other, owing to their negative charges; these arose from WPU aggregation at the air–water interface during the coarsening process.^43^ The SP-loaded samples exhibited significantly lower average bubble sizes than WPU–SP0 15 min after foaming, i.e., decreased significantly to 22.27, 21.56, and 15.93 μm for WPU–SP2, WPU–SP4, and WPU–SP6, respectively (Figure 3J–L). WPU–SP2 and WPU–SP4 exhibited a larger bubble size than WPU–SP0 12 h after foaming (362.39 and 319.52 μm, respectively) and evolved into a regular ball shape (Figure 3M–O). Other than WPU–SP2/4, SP of WPU–SP6 dispersed in the aqueous media and thus adhered to the large, pure WPU bubble interfaces, which further increased the mechanical properties and rigidity of the air–water interface, resulting in the bubbles expanding to form rock-shaped bubbles after 6 h of aging (Figure 3P). On the contrary, the SP concentrations found in WPU–SP2 and WPU–SP4 were insufficient to hinder the motion of bubbles; nevertheless, they did elevate the continuous phase’s viscosity and restrict the processes of coarsening and disproportionation.

### 3.3 Rheology of WPU–SP Pickering Foams

**Figure 4:**
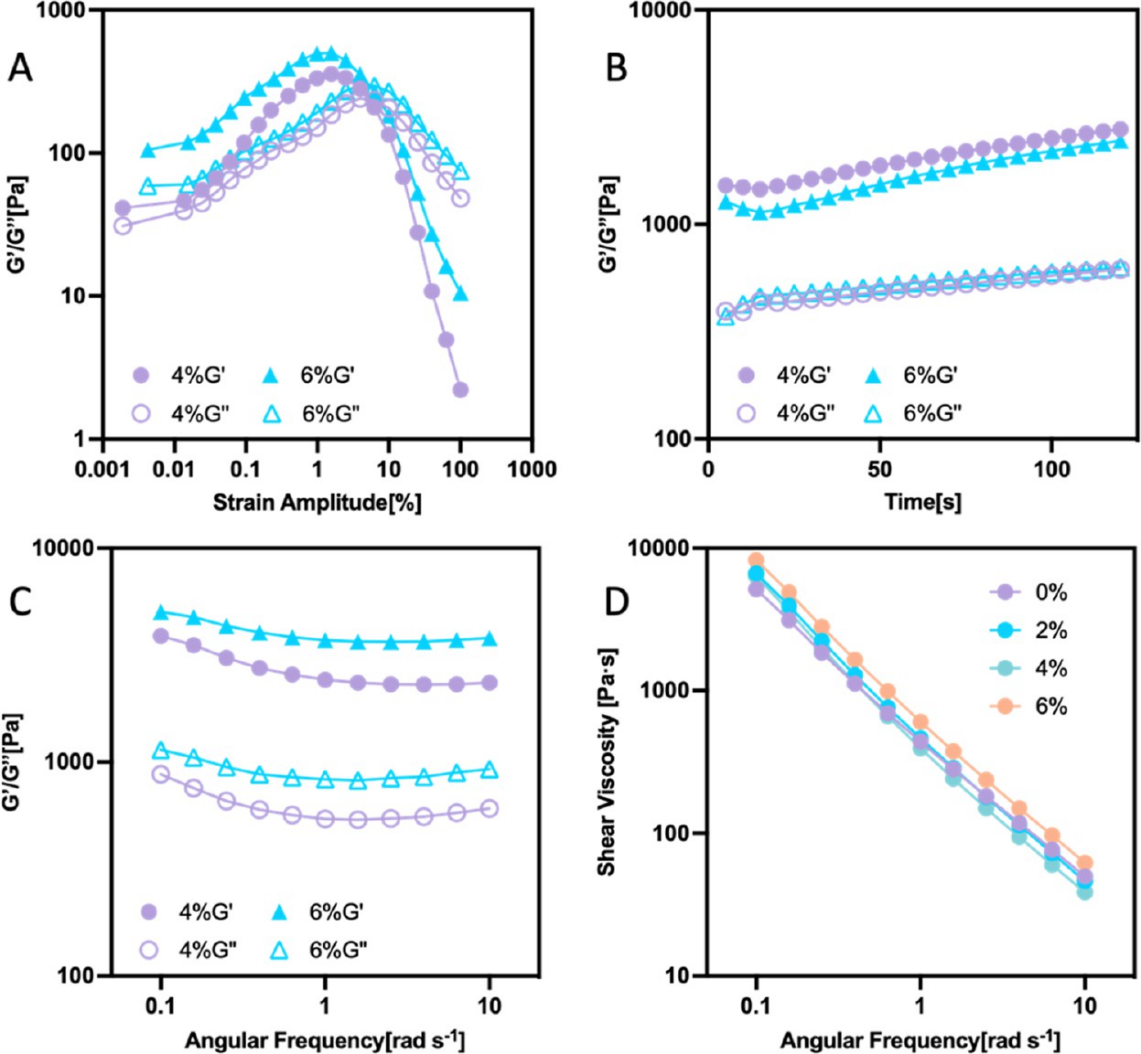
Results of dynamic oscillatory shear test conducted in (A) amplitude sweep, (B) time sweep, and (C) frequency sweep modes to measure the shear viscosity, elastic modulus (*G*′), and viscous modulus (*G*″) of WPU–SP0, WPU–SP2, WPU–SP4, and WPU–SP6, respectively. (D) Shear viscosity measured in the frequency sweep mode.

To further investigate the stabilization mechanism and rheological properties of the WPU–SP Pickering foams, WPU–SP0/2/4/6 were aged for 15 min and then subjected to dynamic oscillatory shear tests, as described in Section 2.4. The rheological behaviors of SP-loaded Pickering foams in amplitude sweep, time sweep, and frequency sweep modes can reveal the differences between their foam stabilities and foam bubble behaviors during the coarsening and disproportionation processes. After 6 h of aging, WPU–SP6 exhibited rock-shaped bubbles, whereas WPU–SP2 and WPU–SP4 remained regular sphere-like bubbles. The variation in the SP content primarily contributed to the rheological difference. In the amplitude sweep tests, WPU–SP4 and WPU–SP6 showed the same critical strain amplitude (1.56%, Figure 4A). The wet foams exhibited fluid-like properties after the amplitude increased to 6.26%. Under conditions of modest shearing amplitude, all Pickering foams demonstrated viscoelastic behavior since their elastic modulus is greater than their viscous modulus. WPU–SP0 and WPU–SP2 exhibited higher initial viscous modulus than elastic modulus, possibly because the liquid WPU drainage destroyed the elastic state of intact Pickering foam (Figure S5). WPU–SP6 exhibited a higher elastic modulus and viscous modulus than WPU–SP4 in the time sweep and frequency sweep tests (Figure 4B,C and S6–7). During the frequency sweep test at a low strain amplitude (1%), all of the Pickering foams exhibited higher elastic modulus than viscous modulus, consistent with the amplitude sweep results (Figure S5). The observed solid-like behaviors and increased elastic modulus indicate that Pickering foams containing larger amounts of SP have better stability, making them appropriate for long-term storage and potential use as templates for porous film fabrication. WPU–SP6, with the highest SP content, exhibited a higher shear viscosity than the other Pickering foams, i.e., a shear viscosity of 8207 Pa·s at 0.1 rad/s during the frequency sweep. As the shearing speed increased, the shear viscosity of the Pickering foams significantly decreased because their networks of WPU latex particles and SPs were destroyed under high-speed shearing (Figure 4D). WPU–SP6 exhibited the highest initial elastic modulus and viscosity at low-frequency conditions, confirming the formation of a strong network structure in the continuous liquid phase. The strong network influenced the movement of foam bubbles, as dispersal of SPs in the liquid phase resulted in an increase in the number of SPs that were wetted by WPU and in the bridges formed between WPU particles.^42^ These findings confirm the observations made during the WPU–SP6 foam-bubble-coarsening process and indicate that its strong network can retain its original well-dispersed bubble distribution for a long period, making this foam suitable to a template for porous film preparation after drying.

### 3.4 Structure of WPU–SP Composite Porous Films

**Figure 5:**
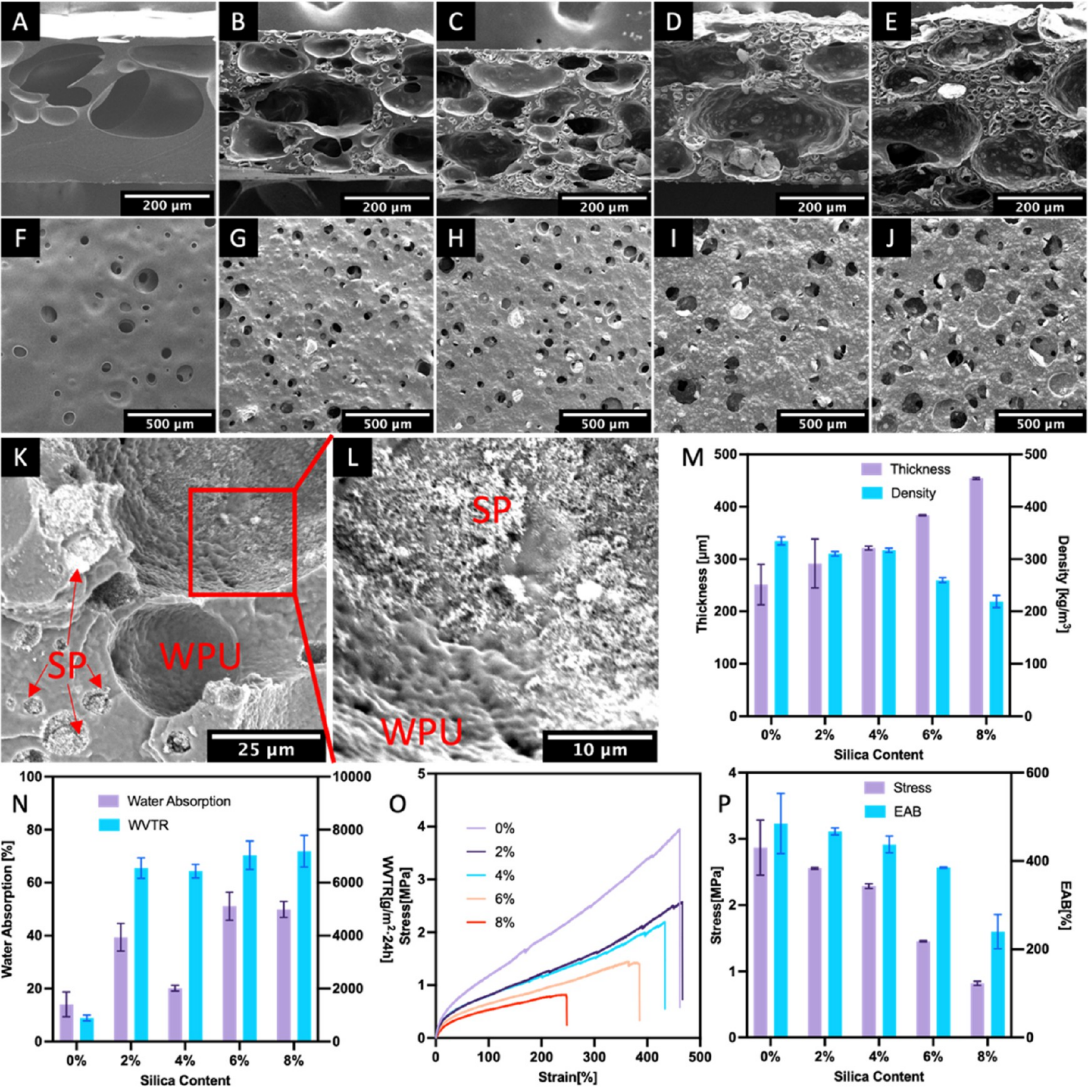
Porous structures of the as-prepared WPU–SP composite films were characterized from a section view (A–E) and surface view (F–J) separately: (A, F) 0 wt %, (B, G) 2 wt %; (C, H) 4 wt %, (D, I) 6 wt %, and (E, J) 8 wt % SP content. The magnified section view (K, L) indicated the interfacial structure assembled from WPU and SPs and the position of SPs after drying. Technical parameters of the WPU–SP composite films were summarized: thickness and density (M), WA and WVTR values (N), and tensile stress and EAB (O, P).

WPU–SP composite porous films were prepared using the WPU–SP Pickering foams with different SP contents as a template, as described in Section 2.5 and Figure 1A. The templated porous structures of WPU–SP Pickering foams were evaluated via FE-SEM. The pure WPU exhibited an uneven distribution of porous structures with open cells (Figure 5A). WPU–SP0 exhibited drainage during the drying process because of the low viscosity of its wet foam. Liquid WPU leaked out owing to gravity and accumulated below the foam layer, which resulted in a dense structure in the lower part of the porous film.^44^ In contrast, the porous films containing SPs exhibited a well-distributed open-cell porous structure (Figure 5B–E). During the heating process to transform the liquid Pickering wet foam film to the dry porous WPU–SP composite film, water-vapor evaporation caused the volume of the liquid film in the continuous phase to decrease. The thin film between the Pickering foam bubbles also becomes thinner over time. The concentration of polyurethane latex particles in the continuous phase gradually increases, and the distance between them decreases until merging. Eventually, all the water evaporating resulted in the formation of the pore wall. Simultaneously, the gas in the foam bubbles expands as a result of heating, acting as a pore-forming agent, which ultimately leads to the formation of an open-cell porous structure regardless of the temperature at which the liquid films are dried (Figure S4).

When comparing the porous films with lower SP contents to those with higher SP contents, it was seen that the latter had more crowded holes. This is due to the presence of more bubbles which are stabilized by WPU and SPs. The WPU–SP holes were much smaller than the pure WPU templated holes. These smaller holes originated from the WPU–SP stabilized bubbles in the continuous phase and occurred even within the area surrounding the larger pure WPU templated holes. This could be observed in the inner wall of the pure WPU holes. The WPU–SP templated holes exhibited no significant change in size. The average bubble size of the WPU–SP6 Pickering foam was 15.93 μm, whereas the size of the WPU–SP templated holes after drying was 15.61 μm. This indicates that the air–water interface of the WPU–SP Pickering foam was reinforced by SPs, which ensured a rigid state for templating. Furthermore, the increase in the SP contents increased the cell openness degree and hole size at the film surface (Figure 5F–J). Particle (WPU latex particles and SPs) aggregation in the dry state occurred (Figure 5K) at the air–water interface of both WPU–SP holes and pure WPU holes in the porous films. The inner wall of most holes was covered with SPs in the aggregated state. Both the bare pure WPU holes and the inner walls without an SP covering exhibited packed WPU latex particles. The magnified view of WPU–SP holes showed that aggregated SPs were adhered to the WPU wall (Figure 5L), confirming the WPU–SP particle assembly structure of the Pickering wet foam discussed above.

The thickness and density data of the porous films with SP loading of 0, 2, 4, 6, and 8 wt % are presented in Figure 5M. SP addition increased the thickness of the porous film. WPU–SP0 exhibited the lowest thickness because of bubble coalescence and rupture. The rigid WPU–SP Pickering foam bubbles retained their shape after water evaporation, although the film thickness decreased. The network formed between WPU and the dispersed SPs in the continuous liquid phase also hindered the drainage process and enhanced the strength of the thin film within foam bubbles. Therefore, with increasing SP content, the thickness of the WPU–SP composite porous film increased, while the density decreased. WPU–SP8 exhibited the highest thickness (454 μm) and lowest density (219 kg/m^3^). The retention of abundant porous structures in the film reduced the moisture transfer resistance and further enhanced the WA and water-vapor permeability (Figure 5N).^44,45^ The pure WPU porous film WPU–SP0 exhibited WA and WVTR values of only 14% and 892 g/m^2^·24 h, respectively, owing to its dense structure. SP addition effectively improved the WA value: the WA values of the WPU–SP6 and WPU–SP8 composite films (51.1 and 49.9%, respectively) were over three times that of the pure WPU film. The WPU–SP composite porous films also exhibited significantly improved WVTR values because of the absence of a dense structure. A higher SP content led to higher water-vapor permeability because abundant WPU–SP holes were retained in the film, which compensated for the loss in water-vapor transmission caused by unfavorable thickness increases. The as-prepared dry film samples were also subjected to a tensile test. Compared with the films with lower SP loadings, those with higher SP loading exhibited higher porosities, which resulted in lower stress performance and EAB values. The WPU porous film with a dense structure had the greatest tensile stress of 4.28 MPa and EAB values of 540%. Owing to their rich porous structures, the WPU–SP6 and WPU–SP8 composite porous films exhibited significantly lower mechanical strengths (1.45 and 0.82 MPa, respectively) than the pure film. The presence of abundant porous structures in the WPU–SP8 film reduced its capacity to withstand intensive stretching, resulting in an EAB of 240%.

### 3.5 Application of WPU–SP Composite Porous Films

**Figure 6:**
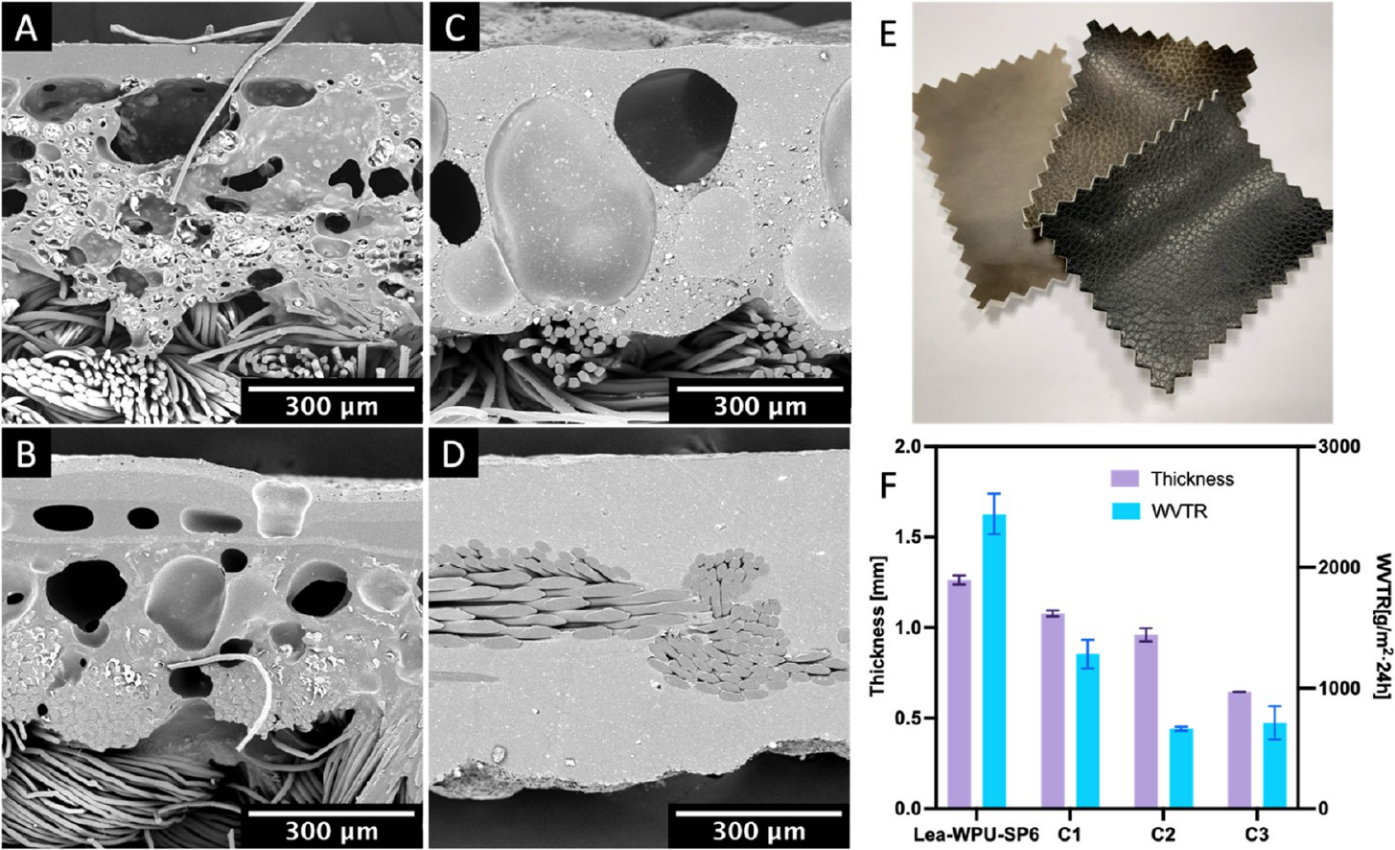
Sectional views of synthetic leather samples: (A) Lea-WPU–SP6; (B–D) commercial synthetic leather samples C1, C2, and C3. (E) Graphs of lab-made synthetic leather templated from releasing papers with various aesthetic patterns. (F) Comparison of the thicknesses and WVTR values of the Lea-WPU–SP6 and commercial samples (C1, C2, and C3).

A lab-made synthetic leather with the WPU–SP Pickering foam-templated porous film as the midlayer was prepared via a four-step method, as described in Section 2.5 and Figure 1B. Moreover, lab-made synthetic leather samples with different surface patterns, namely, lychee, lambskin, and frosted patterns, were successfully prepared using typical patterned releasing papers (Figure 6E). The lab-made samples featured a sandwich structure, including a dense surface layer, porous middle layer, and fabrics substrate (Figure 6A). The middle-layer templated from WPU–SP Pickering foam enabled effective wetting of the fabrics. Small-sized WPU–SP holes and large-sized pure WPU holes can be observed in the midlayer, comparable to the dry WPU–SP porous film sample. The cross-sectional view of commercial synthetic leather samples (C1, C2, and C3), composed of polymer resins and textile fabrics, was assessed using FE-SEM to analyze their porous structures. The double porous layers of C1 (Figure 6B) meant two-step foam layer coating is needed to achieve acceptably thick and porous structures. The polymer layer of C2 exhibited large dimension compact porous structures (Figure 6C), while C3 consisted of fabrics and dense polymeric coatings without any porous structures. The water affinity including WVTR and WA of all of the lab-made synthetic leather samples was assessed as shown in Figure S8A,B. When the hydrophobic SP increases, samples such as Lea-WPU–SP6/8, exhibited superior WVTR compared to the Lea-WPU–SP0 without SP particles, which has been related to the enhanced porous structure at high SP particles. However, when the SP concentration is too high, the WVTR (Lea-WPU–SP8) would decrease, likely due to a high viscosity of the medium, which decreased the performance of the blade-coating. Compared with C1 and C2, the Lea-WPU–SP6 sample exhibited a higher leather thickness (1.26 mm), a comparable intermediate porous-layer thickness (390 μm), and a higher WVTR value (2442 g/m^2^·24 h). Lea-WPU–SP6 also demonstrated excellent water-vapor permeability, which was 6.78 times that of the industrial standard (QBT 1646–2007 and JIS.K6601–1995, i.e, 360 g/m^2^·24 h), owing to it having an WPU–SP Pickering foam-templated porous film as its intermediate layer. The compact porous structures of C1 and C2 and the thin but dense structure of C3 limited their water-vapor permeability, although these samples also met the industry requirement.

## 4 Conclusions

WPU–SP Pickering foams were successfully produced by adding hydrophobic SPs to WPU at a concentration of up to 8 wt % via homogenization. The stability of this binary particle-stabilized wet foam system was studied in samples with varying SP contents. SPs stabilized the air–water interface of foam bubbles with WPU latex particles by adhering to the inner wall of the foam bubbles. SP addition increased the rigidity of the WPU–SP Pickering foam bubbles and thus improved foam stability. The hydrophobic interaction between the PU chain backbone and SPs also allowed the SPs dispersed in the continuous liquid phase to bridge WPU latex particles to form a strong network structure that stabilized the foam systems. The robust network architecture increased the liquid phase viscosity, restricted foam drainage, and restricted foam bubble movement. This limited the coalescence of foam bubbles, which further improved the structural stability of the WPU–SP Pickering foam. The wet foam with an SP content >6 wt % remained stable over 1 month. The SPs migrated into the continuous phase during the foam aging process and covered the existing foam bubbles, endowing the bubbles with a rock-like shape, which allowed them to expand further. The WPU–SP films templated from the WPU–SP Pickering foam exhibited a good porous structure, dimensional stability, excellent WA values, and water-vapor permeability. The WPU–SP composite porous film with an SP content of 6 wt % was successfully applied as an intermediate porous layer for fabricating synthetic leather, which exhibited substantial thickness and wetting on fabrics. Moreover, the lab-made synthetic leather exhibited excellent water-vapor permeability, which was 6.78 times that of the industrial standard. Overall, the Pickering foam template method provides an efficient, clean, and sustainable strategy for fabricating novel synthetic leather with high water-vapor permeability.

## Supporting Information Available

Water contact angle of hydrophobic fumed silica nanoparticles (HDK H18); shelf life of WPU–SP2/4 Pickering foam in 30 days after foaming; rheology test for WPU–SP0/2/4/6 Pickering foam under amplitude sweep, time sweep, and frequency sweep; liquid and foam volume change of WPU–SP0/2/4/6 Pickering foam during the 7-day aging process (PDF)
