# Supplementary material for: Porous Waterborne Polyurethane Films Templated from Pickering Foams for Fabrication of Synthetic Leather
Source: Langmuir. 2024 Feb 22;40(9):4751–61. doi: 10.1021/acs.langmuir.3c03514 (PMC10919083; doi:10.1021/acs.langmuir.3c03514)
Supplement: Supplementary file 1 — la3c03514_si_001.pdf [file la3c03514_si_001.pdf]

Supporting Information

Porous Waterborne Polyurethane Films Templated from Pickering Foams for Fabrication of Synthetic Leather

Zhenghao Shi[a], Yifeng Sheng[a], Jianhui Wu[b], Jiwei Cui[c], Wei Lin[b,], To Ngai[a,*]*

[a]Department of Chemistry, The Chinese University of Hong Kong, Shatin, N. T., Hong Kong, 999077, China.

[b]Department of Biomass and Leather Engineering, Key Laboratory of Leather Chemistry and Engineering of Ministry of Education, Sichuan University, Chengdu, 610065, China.

[c]Key Laboratory of Colloid and Interface Chemistry of the Ministry of Education, School of Chemistry and Chemical Engineering, Shandong University, Jinan, Shandong 250100, China.

* Corresponding authors: tongai@cuhk.edu.hk & wlin@scu.edu.cn

Keywords: Pickering foam, Waterborne polyurethane, Silica particles, Synthetic leather, Clean production

Table of Contents

Number of pages: 10

Number of figures: 8

Number of tables: 1

Figure S1. The water contact angle of SP (H18) S3
Figure S2. Shelf life of WPU-SP2Pickering foam S4
Figure S3. Shelf life of WPU-SP4 Pickering foam S4
Figure S4. Porous structures of WPU-SP6 composite films dried at different temperatures........S5
Figure S5. Rheology test for WPU-SP0/2/4/6 under amplitude sweep..S6
Figure S6. Rheology test for WPU-SP0/2/4/6 under time sweep S7
Figure S7. Rheology test for WPU-SP0/2/4/6 under frequency sweep..S8
Figure S8. WVTR and water absorption of the Lea-WPU-SP0/2/4/6/8 ....S9
Table S1. Wet foam aging performance for WPU-SP0/2/4/6 Pickering foam samples.............S10

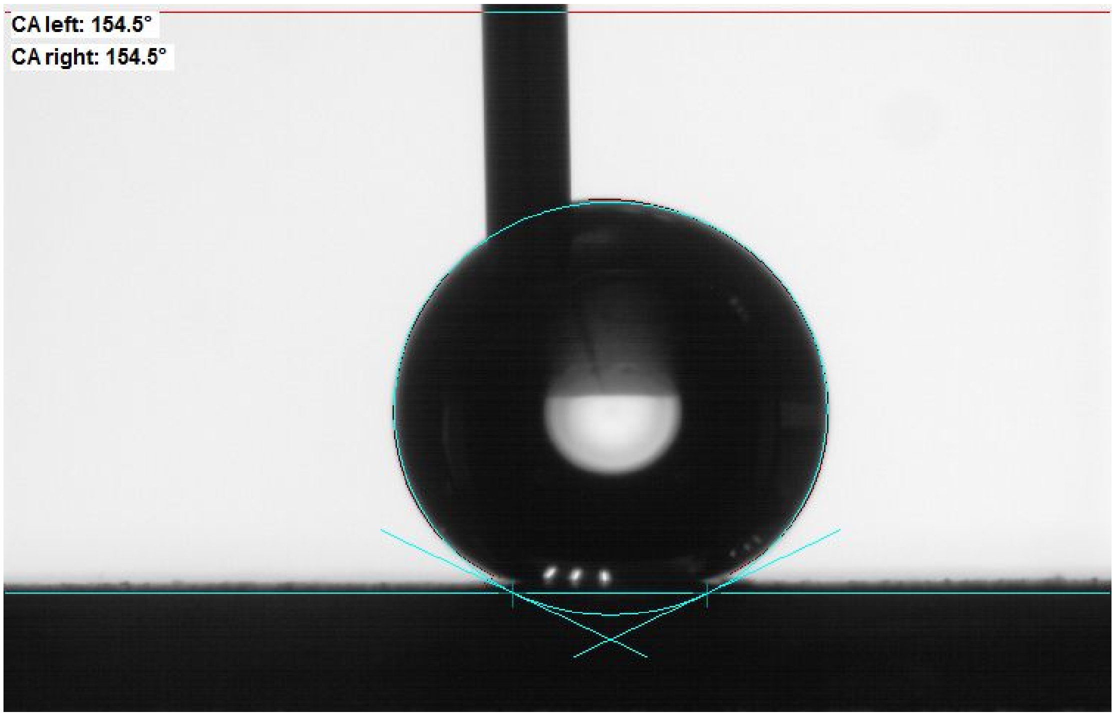

Figure S1. The water contact angle of SP (H18) is at 154.5°.

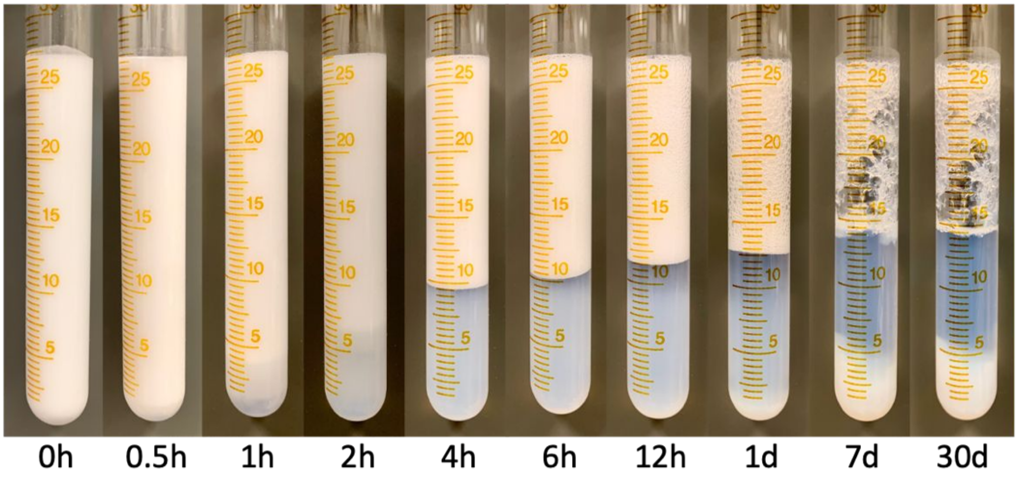

Figure S2. Shelf life of WPU-SP2Pickering foam in 30 days after foaming.

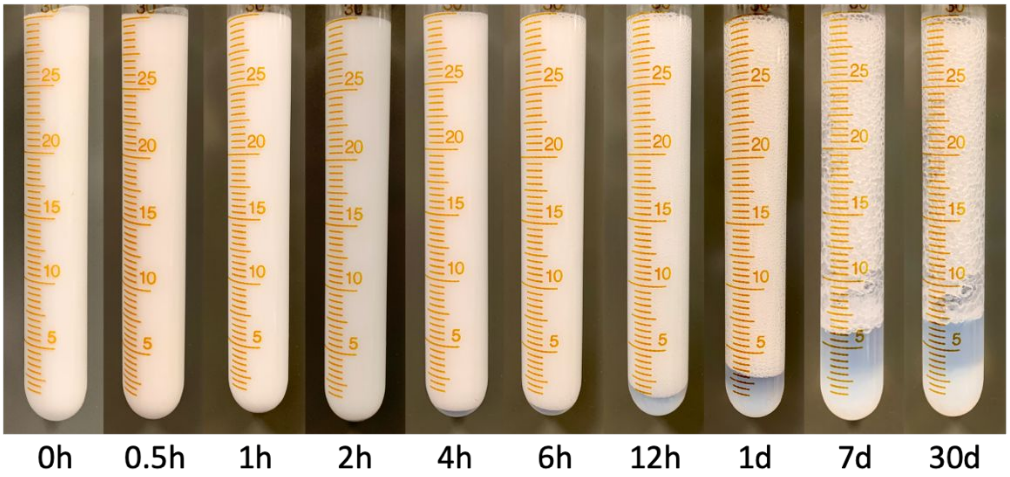

Figure S3. Shelf life of WPU-SP4 Pickering foam in 30 days after foaming.

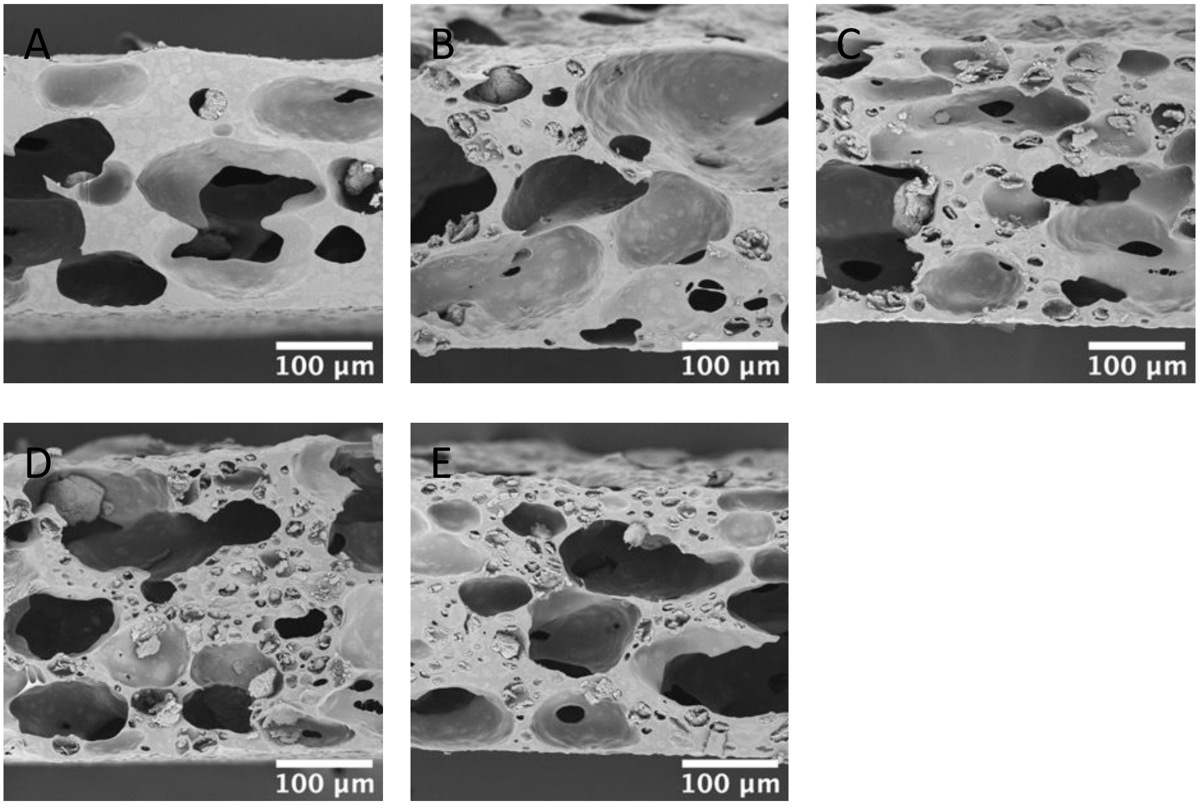

Figure S4. Porous structures of a typical as-prepared WPU-SP6 composite films dried at (A) room temperature, (B) 40°C; (C) 60°C, (D) 80°C, and (E) 100°C as measured from section view by SEM.

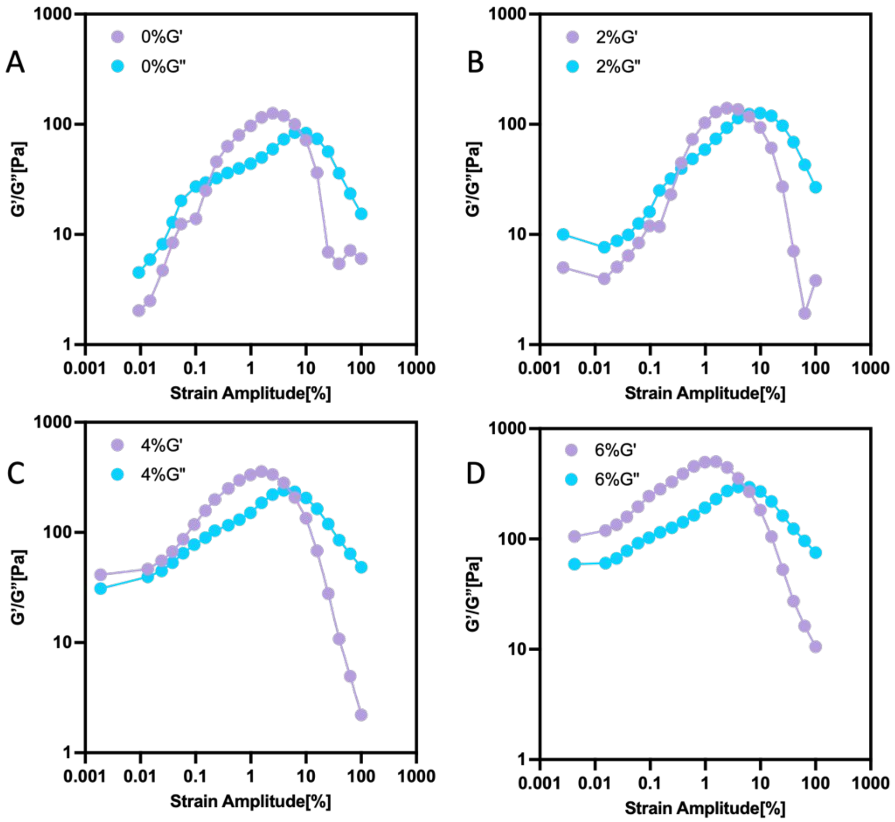

Figure S5. Rheology test for WPU-SP0/2/4/6 under amplitude sweep.

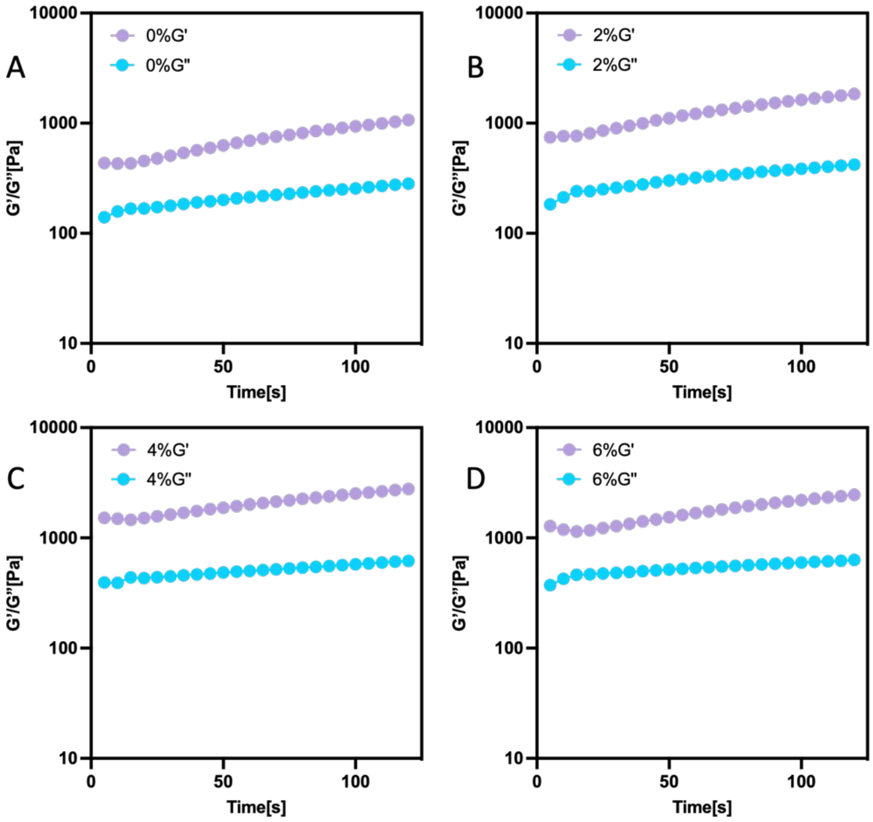

Figure S6. Rheology test for WPU-SP0/2/4/6 under time sweep.

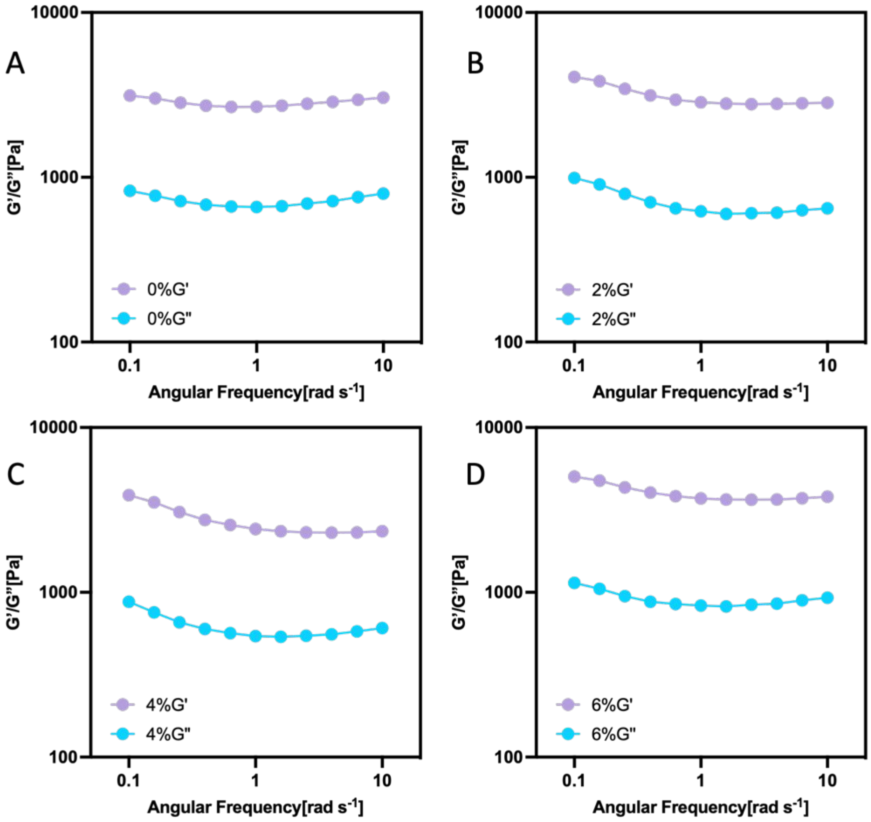

Figure S7. Rheology test for WPU-SP0/2/4/6 under frequency sweep.

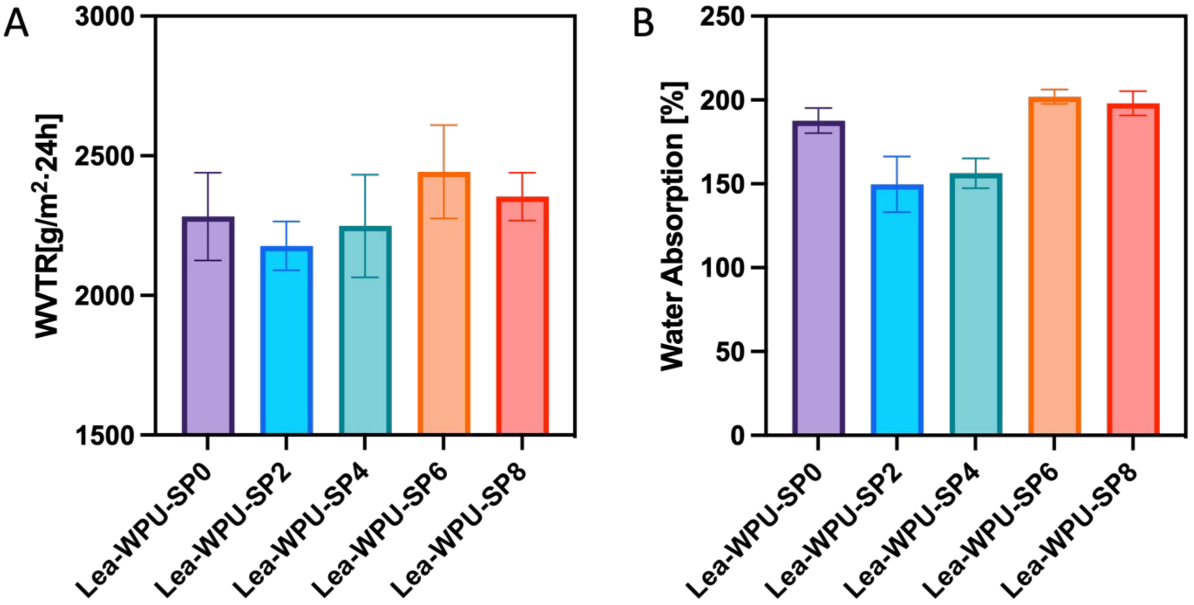

Figure S8. The effect of concentration of SP on the (A) WVTR and (B) water absorption of the Lab-made synthetic leather samples Lea-WPU-SP0/2/4/6/8.

Table S1. Wet foam aging performance evaluation in foam/liquid volume change for WPU-SP0/2/4/6 Pickering foam samples.

	Time	1h	2h	4h	6h	12h	24h	7d
WPU-SP0	Total Volume[mL]	27	27	26	25.5	24	18	17
	Foam Volume[mL]	20	17	10	9	7	0.5	0
	Liquid Volume[mL]	7	10	16	16.5	17	17.5	17
	Foam Percent	74%	63%	37%	33%	26%	2%	0%
	Liquid Percent	26%	37%	59%	61%	63%	65%	63%
WPU-SP2	Total Volume[mL]	27	27	27	27	27	27	27
	Foam Volume[mL]	23	21	17.5	17	15.5	14.5	12.5
	Liquid Volume[mL]	4	6	9.5	10	11.5	12.5	14.5
	Foam Percent	85%	78%	65%	63%	57%	54%	46%
	Liquid Percent	15%	22%	35%	37%	43%	46%	54%
WPU-SP4	Total Volume[mL]	30	30	30	30	30	30	30
	Foam Volume[mL]	30	30	29.5	29.3	28.5	27	24
	Liquid Volume	0	0	0.5	0.7	1.5	3	6
	Foam Percent	100%	100%	98%	98%	95%	90%	80%
	Liquid Percent	0%	0%	2%	2%	5%	10%	20%
WPU-SP6	Total Volume[mL]	26	26	26	26	26	26	26
	Foam Volume[mL]	26	26	26	26	26	26	25.7
	Liquid Volume[mL]	0	0	0	0	0	0	0.3
	Foam Percent	100%	100%	100%	100%	100%	100%	99%
	Liquid Percent	0%	0%	0%	0%	0%	0%	1%
